# Unusual thoracic manifestation of metastatic malignant melanoma

**DOI:** 10.4103/0970-2113.63615

**Published:** 2010

**Authors:** K. Manu Mohan, K. Gowrinath

**Affiliations:** *Department of Pulmonary Medicine, Kasturba Medical College, Manipal, Karnataka, India*

**Keywords:** Malignant melanoma, pleural effusion, pleurodesis, vincristine

## Abstract

Massive pleural effusion due to metastatic malignant melanoma is rare. We report a case of bilateral (massive on left side) pleural effusion as a metastatic manifestation of cutaneous malignant melanoma. In our case, successful outcome of pleurodesis with vincristine is significant as this agent is rarely used.

## INTRODUCTION

The incidence of melanoma is increasing worldwide when compared to most other neoplasms even in the countries where its incidence was previously low.[1] The White population have approximately 10 times greater risk of developing cutaneous malignant melanoma than the Asian, Hispanic or Black population.[2] In India, the exact incidence of malignant melanoma is not known and is still regarded as an uncommon neoplasm. Most primary cutaneous melanomas are cured by surgical excision but metastatic spread continues to occur in 30% cases, most often to the lung.[3] Pleural effusion is an infrequent presentation of metastatic malignant melanoma.[4] We present a case of bilateral pleural effusion as an unusual metastatic manifestation of malignant melanoma of scalp.

## CASE REPORT

**Figure 1:**
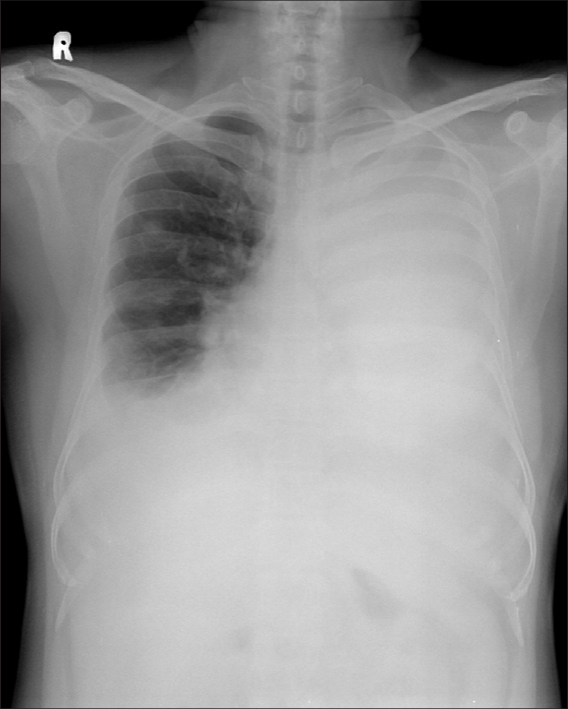
Chest radiograph showing bilateral pleural effusion, massive on left side

**Figure 2:**
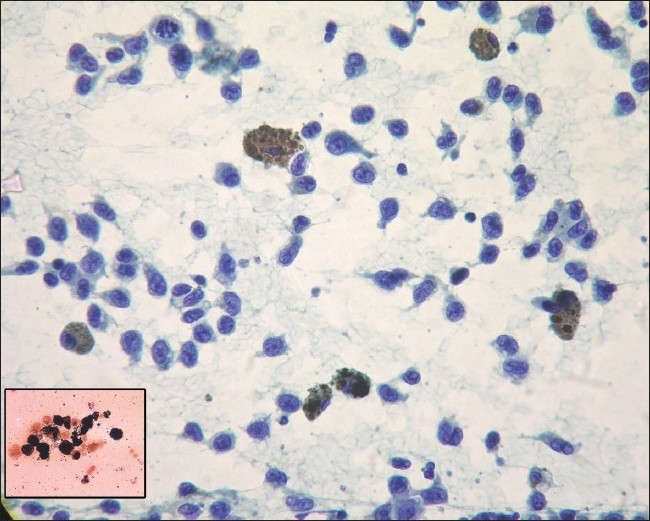
Pleural fluid smear showing malignant cells, some of them containing melanin pigment in their cytoplasm. (Papaniculaou stain × 400). Inset showing melanin pigment staining positive with Fontana Masson's stain. (Fontana Masson's × 400)

A 35-year-old male nonsmoker was referred to us for evaluation of cough and breathlessness of two weeks duration. He had malignant melanoma (nodular type) of scalp eight months ago but did not undergo surgery. There was no history of any other significant illness. On physical examination, there was biopsy scar over the occipital region and naevi all over the body. There was no digital clubbing or significant lymphadenopathy. Chest examination showed features of bilateral pleural effusion. Abdominal examination revealed nodular hepatomegaly. Other systems including eyes were clinically normal. A chest radiograph [Figure 1] showed bilateral pleural effusion, massive on left side. Upon diagnostic thoracentesis on both sides, dark colored pleural fluid was aspirated and its cytological examination [Figure 2] showed malignant cells, some of them contained variable amount of melanin pigment in their cytoplasm. The cytoplasmic pigment stained black with Fontana Masson's cytoplasmic stain, highlighting the presence of cytoplasmic melanin within the tumor cells.

A cytological diagnosis of metastatic malignant melanoma was made. Ultrasound examination of abdomen showed hepatomegaly. Pleural effusion re-accumulated rapidly despite repeated therapeutic pleurocenteses. Tube thoracostomy was done on left side for drainage of the pleural fluid. After three days, the left lung expanded completely showing patchy opacities over upper lobe and the daily pleural fluid drainage was less than 100ml. The pleurodesis was attempted with 2 mg of vincristine diluted in 50 ml of normal saline through thoracostomy tube after clamping it distally. Intramuscular tramadol was given to reduce the pain after the pleurodesis. The chest tube was unclamped after two hours and removed the next day as pleural drainage was less than 50ml. Two months later, the patient presented with cough and right sided pleuritic chest pain of one week duration. Cough was occasional and nonproductive.

**Figure 3:**
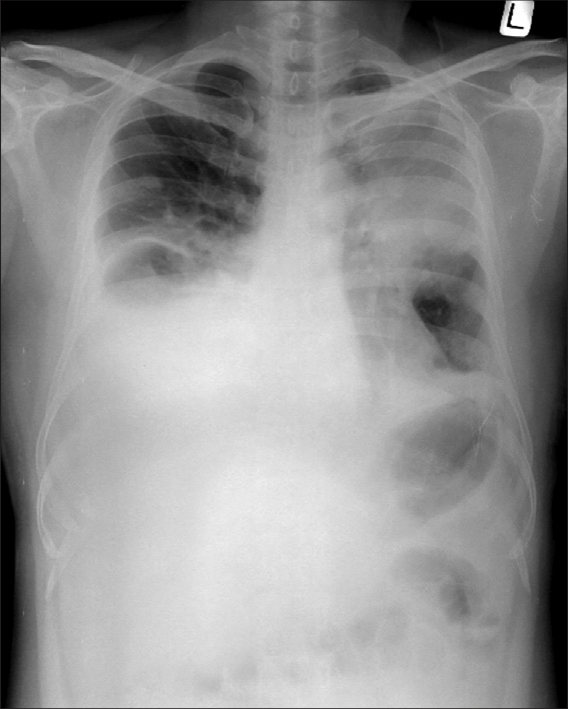
Chest radiograph showing increase in the size of left upper lobe mass with underlying collapse and increased pleural effusion on right side as compared to the previous chest radiograph

Chest radiograph [Figure 3] showed increase in size of left upper lobe mass and pleural effusion on right side. Cytological examination of pleural fluid showed metastatic malignant melanoma. The chest pain improved with the oral morphine and the pleurodesis was done for right sided pleural effusion using vincristine. After discharge, patient did not come for review and was lost to follow up.

## DISCUSSION

Metastatic malignant melanoma constitutes about 5% of all secondary malignancies of lung.[5] Previously, in a study of 130 cases of malignant melanoma with thoracic involvement, only 2% had pleural effusion; one of them with massive effusion.[6] Only a few reports of massive pleural effusion due to metastatic malignant melanoma are available. A thoracoscopic pleural biopsy proven metastatic malignant melanoma causing left sided massive pleural effusion was reported in a white man who had malignant melanoma of scalp 14 years ago.[7] In our patient, the diagnosis was made through cytological examination of pleural fluid. Our patient had increased risk of getting malignant melanoma as he had naevi all over the body.[8] Recently, another report of bilateral pleural effusion with ascites as a metastatic manifestation of malignant melanoma is reported.[9] We are not aware of any previous Indian report of bilateral pleural effusion due to metastatic malignant melanoma. Malignant melanoma is an aggressive neoplasm and in our patient, pleural involvement occurred eight months after its cutaneous presentation over the scalp. In malignant effusions, pleurodesis is advocated for palliation as the survival is limited.[10]

Talc is the most effective sclerosing agent available for pleurodesis and bleomycin is considered as an alternative sclerosant because of its moderate efficacy rate.[11] Iodopovidone, a topical antiseptic was reported to be a safe, inexpensive and effective alternative to achieve pleurodesis.[12] We have been using vincristine as a cheaper alternative to bleomycin even though it was not listed as a sclerosing agent.[13] In our institute, vincristine was found to be a safe and effective sclerosing agent for pleurodesis.[14] Pleurodesis with vincristine was successful in our patient as the left sided pleural effusion did not recur even after two months. In conclusion, we have reported a bilateral pleural effusion, massive on left side as a rare presentation of metastatic malignant melanoma. Since vincristine is rarely used for pleurodesis of malignant effusion, further multicentre evaluation of its use is required before recommending it as a cheaper alternative to other agents used for pleurodesis.
